# Surgical stabilization of the ipsilateral scapula and rib fractures using the mirror Judet approach: a preliminary result

**DOI:** 10.1186/s12891-021-04991-2

**Published:** 2022-01-31

**Authors:** Chang-Han Chuang, Chin-Kai Huang, Chia-Ying Li, Ming-Hsien Hu, Pei-Yuan Lee, Po-Ting Wu

**Affiliations:** 1 Department of Orthopaedic Surgery, Show Chwan Memorial Hospital, 524 Sec. 1 Chung-Shan Rd., Changhua, 500 Taiwan; 2 Department of Life Sciences, National Chung Hsing University, Taichung, Taiwan; 3 Ph.D. Program in Translational Medicine, National Chung Hsing University, Taichung, Taiwan; 4 Department of Orthopedics, National Cheng Kung University Hospital, College of Medicine, National Cheng Kung University, Tainan, Taiwan; 5 Department of Surgery, Show Chwan Memorial Hospital, Changhua, Taiwan; 6 Department of Biomedical Engineering, National Cheng Kung University, Tainan, Taiwan; 7 College of Nursing and Health Sciences, Da-Yeh University, Changhua, Taiwan; 8 Department of Orthopedics, College of Medicine, National Cheng Kung University, 1 University Road, Tainan, 701 Taiwan; 9 Department of Orthopedics, National Cheng Kung University Hospital Dou-Liou branch, College of Medicine, National Cheng Kung University, Yunlin, Taiwan; 10 Medical Device Innovation Center, National Cheng Kung University, Tainan, Taiwan

**Keywords:** Mirror Judet approach, Scapula, Ribs, Fracture fixation

## Abstract

**Background:**

We report our preliminary results using a single approach, the mirror Judet approach, for patients with both ipsilateral scapula and multiple rib fractures.

**Methods:**

Five consecutive patients [median age: 56 years (range: 44 ~ 60)] with ipsilateral scapula and multiple rib fractures that met the surgical indications were retrospectively reviewed. A single approach, the mirror Judet approach, was used for surgical stabilization of the scapula and targeted rib fractures. Thoracoscopic surgery was performed first for management of associated lung lesions and marking the targeted rib. All patients received the same rehabilitation protocol and a minimum 12-month follow-up.

**Results:**

All surgically-fixed fractures eventually united without malunion. No complaints of intercostal neuralgia, infection, or other complications were seen. The mean range of motion in the injured shoulder returned to at least 90% of the contralateral side range. The mean Disabilities of the Arm, Shoulder, and Hand score at the 12th month was 2.0 (range: 0-7). All patients were able to return to their previous work.

**Conclusion:**

The mirror Judet approach allows for the surgical stabilization of the ipsilateral scapula and multiple rib fractures using the same approach and provides acceptable functional outcomes in well-selected patients.

**Level of evidence:**

Level IV.

## Background

Scapula fractures are uncommon [1]. Be that as it may, it has been reported that rib fractures have a high co-incidence (up to 52.9%) with scapula fractures in a retrospective study using a national trauma database [2]. Until now, there is still no clear surgical indication for rib fractures, despite increasing evidence showing the benefits of surgical fixation for chest wall injuries [3–7]. However, for a patient presenting a surgically-indicated scapula fracture [8] and multiple rib fractures, simultaneous surgical fixation of both fractures can be considered in certain cases.

The surgical approach is essential for both scapula fractures and rib fractures. Many different surgical approaches with individual benefits and indications have been proposed in the literature [8, 9]. To our best knowledge, there is no single approach for both ipsilateral scapula and multiple rib fractures. Therefore, we report our preliminary results using a single approach, the mirror Judet approach, for patients undergoing osteosynthesis for both ipsilateral scapula and multiple rib fractures.

## Methods

### Patient population

We retrospectively reviewed the medical records of patients who were treated using the mirror Judet approach from Jul. 2016 to Oct. 2019. The study protocol was approved by the Institutional Review Board of the senior author’s hospital (No.:1090705). Five patients [two males, three females; median age: 56 year-old (range: 44 ~ 60)] had been followed-up for at least 12 months. Our surgical indications for scapula fractures were: (1) medial/lateral displacement ≥ 20 mm; (2) angular deformity ≥ 45°; (3) glenopolar angle (GPA) ≤ 22°; (4) medial/lateral displacement ≥ 15 mm and angulation ≥ 30°; (5) glenoid articular gap >4 mm; and, (6) double disruption of the superior shoulder suspensory complex with a displacement ≥ 10 mm [8, 10]. The indications for surgical stabilization of rib fractures (SSRF) were flail chest, multiple (more than three) rib fractures with bicortical displacement and intolerable pain, and surgical intervention for other pulmonary lesions, such as pulmonary laceration or hemothorax [7, 9].

### Indication and contraindication of mirror Judet approach

The indications using the mirror Judet approach for ipsilateral scapula and rib fixation included: (1) fresh fractures, less than two weeks; (2) scapula fractures AO/OTA 14F0.B, F1.B or B, whose main fixation relied on lateral border fixation with or without a simple glenoid fossa fragment; (3) ipsilateral posterior and/or lateral fractures from the fourth to tenth rib. Contraindications to this approach were: (1) delayed (more than two weeks) or corrective surgery needed aggressive soft tissue release; (2) fixation of scapula without involvement of body lateral border or inferior glenoid fossa; (3) anterior rib fractures, not ipsilateral rib fractures or fractures not involving the fourth to tenth rib; and, (4) glenoid fossa comminuted fractures which complete arthrotomy for visual assessment of intra-articular reduction is always needed. The complete posterior shoulder arthrotomy may be compromised by the mirror Judet approach due to limited access to the posterior shoulder capsule. For intra-articular comminuted fractures, a conventional Judet incision with or without modification should be considered [8, 11, 12].

### Surgical technique

**Fig. 1:**
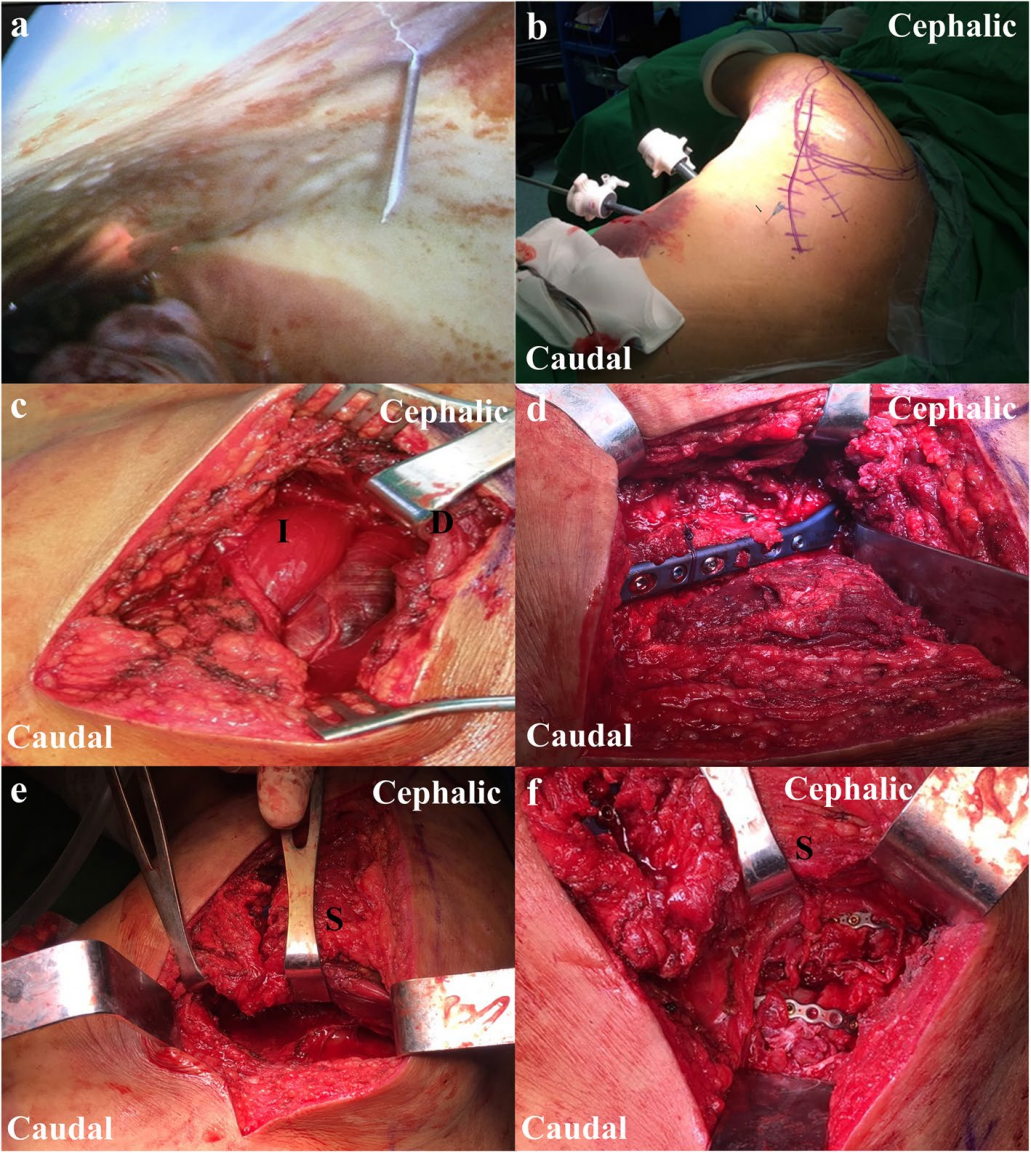
After the thoracoscopic examination and intervention, the fracture site of the targeted rib in the planned fixation was marked with a needle under thoracoscopic vision (**a**). The vertical limb of the mirror Judet incision was marked along the lateral border of the scapula to its lower tip, and then extended to the previously marked needle (arrowhead) to form a lazy L-shaped incision (**b**). The deep approach was taken through the interval between the infraspinatus and teres minor muscle to expose the fracture in the scapular neck or lateral border (**c**). Following the anatomical reduction and rigid fixation of the scapula (**d**), the approach continued down to the oblique limb of the mark for the planned rib fixation. Elevating the scapula is necessary if the targeted rib is higher than the seventh rib with the posterolateral fracture pattern (**e**). The deep approach for rib fracture is similar to the modified muscle-sparing posterolateral thoracotomy approach. Then, the targeted ribs were under direct vision, which facilitated surgical fixation (**f**). I, infraspinatus; D, deltoid; S, scapula

**Fig. 2:**
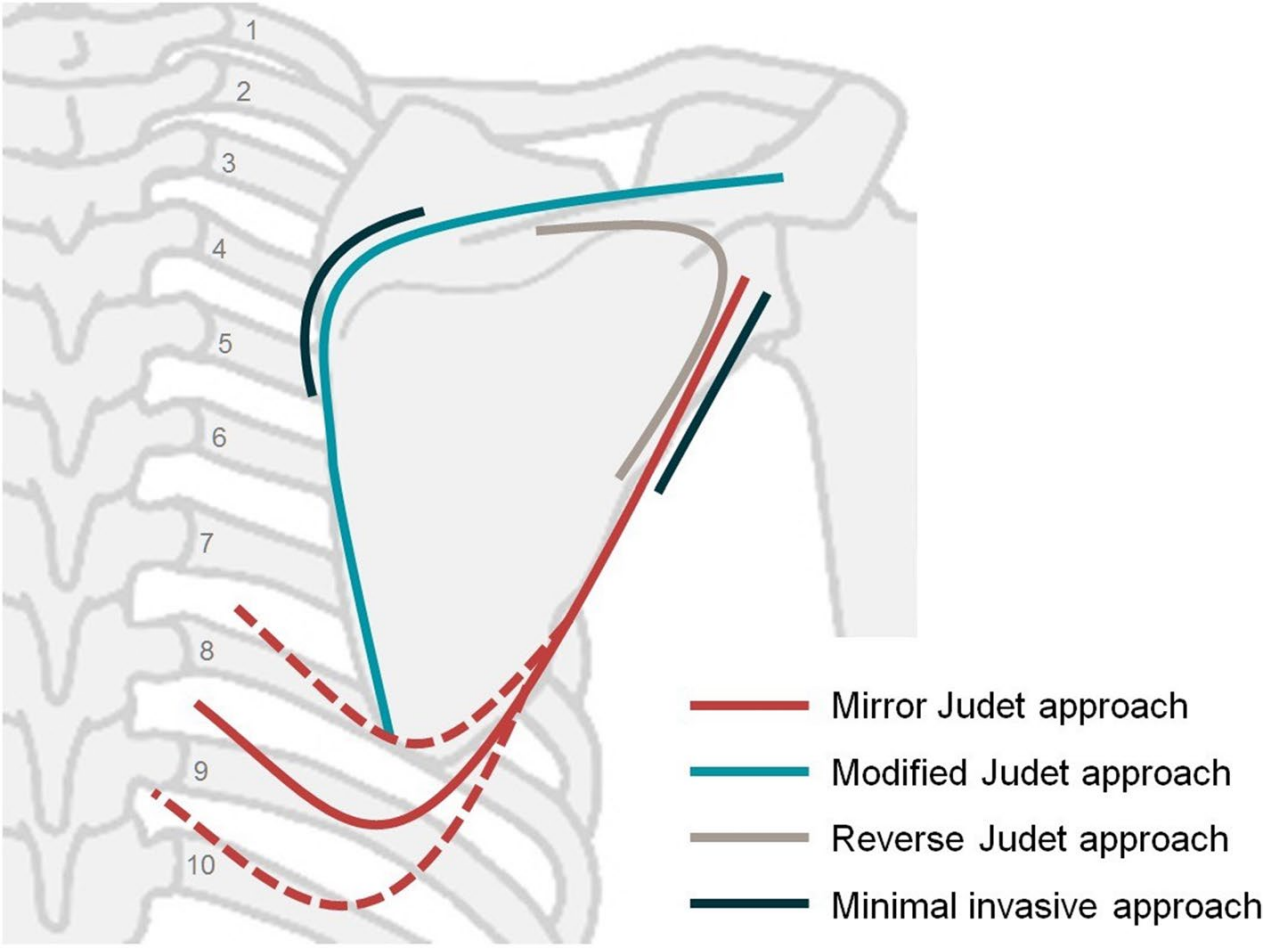
Illustrations of the mirror Judet approach, modified Judet approach [13], minimal invasive approach [14], and reverse Judet approach [15]

The patients were positioned in the lateral decubitus position utilizing a beanbag, with all their pressure points well-padded. The entire forequarter was prepared and draped; however, the ipsilateral arm was drape-free, which aided the intraoperative manipulation of the injured upper extremity and the leaning flexibility of the body (Fig. 1b). Thoracoscopic surgery was performed first by a thoracic surgeon for treatment of hemothorax or/and pneumothorax. Under thoracoscopy, blood and clots were removed. Electrocautery was used for hemostasis of lung parenchyma or the chest wall. Air leaks in the lungs were sealed by endo-stapler, if pneumothorax existed. The fracture site of the targeted rib in the planned fixation was marked with a needle under thoracoscopic vision (Fig. 1a). After the thoracoscopic procedure was completed, a chest tube was inserted. The incision of the mirror Judet approach was designed as a lazy L-shape, beginning from the inferior side of the glenoid, along the lateral border of the scapula to the lower tip, and then extending to the previously marked needle, usually an oblique limb (Fig. 1b and Fig. 2). The vertical limb along the scapula lateral border was incised first. The dissection was taken down to the deltoid fascia, which was divided in line with the deltoid fiber at its inferior edge. The deltoid was retracted cephalad and laterally, revealing the fascia overlying the infraspinatus and teres minor (Fig. 1c). Blunt dissection was used down to the lateral border between the infraspinatus and teres minor, exposing the fracture site. Care was taken in retracting the infraspinatus to avoid tethering or injuring the suprascapular nerve. Furthermore, the ascending branch of the circumflex scapular artery encountered at the lateral border approximately five to six cm below the glenoid rim [16] could be cauterized if necessary [14]. After the fracture site was well exposed, a pair of pointed or two-blunt reduction forceps was used to reduce the displaced lateral border fragments. For a glenoid neck fracture, the reduction technique using a Schanz pin for lateral mobilization of the cephalad fragment and shoulder hook for medial mobilization of caudal fragment is useful [14]. Once anatomical reduction of the fracture site was confirmed, stabilization was achieved with an anatomical locking neutralization plate (Acumed, Hillsboro, Oregon; Fig. 1d).

The incision then continued down to the oblique limb for the planned rib fixation. There is no consensus about the number of ribs that need to be stabilized in treating multiple rib fractures. Usually, it is suggested that the most displaced ribs are the targets for fixation. In our retrospective study, two to four most displaced continuous ribs were repaired, usually the fourth to eighth ribs. Our approach was similar to the modified muscle-sparing posterolateral thoracotomy approach. The safe entry was the so-called triangle of auscultation, bordered by the trapezius superiorly, the latissimus distally, and the scapula medially. Elevating the scapula is necessary if the targeted rib is higher than seventh rib [17] and presents a posterolateral fracture pattern (Fig. 1e). Subperiosteal release of the scapula underlying attachment was taken from the lower tip, and sometimes partial release of the rhomboid major muscle (usually less than one-third attachment to avoid scapulothoracic dyskinesis) [18] would be helpful for better access. Then, the scapula could be gradually elevated using the broad retractor for access to the fourth to sixth ribs. If the scapula fracture is too comminuted or an osteoporotic fracture, we suggest rib fixation prior to scapula fixation. The elevation of the scapula relies on rigid fixation. The blunt muscle splitting could be taken carefully in the latissimus muscle for ribs lower than the seventh rib, serratus anterior muscle for lateral rib fractures, and rhomboid major muscle for posterior rib fractures. Muscle cutting should be avoided. Sometimes, the addition of a percutaneous stabbing incision is helpful for more caudal or anterior fixations. Once the targeted ribs were identified, reduction could be easily achieved using pointed or blunt reduction forceps in simple fractures (Fig. 1f). For a comminuted fracture or flattened-shape fracture site, restoration of alignment should be achieved instead of anatomical reduction. We prefer a 2.4 mm locking reconstruction plate, for osteosynthesis of rib fractures, with at least six cortices of purchase on each side of the fracture. When drilling, real-time arthroscopy monitoring is suggested to avoid lung injury due to over-drilling. The deep muscle fascia and small muscle tear during manipulation could be repaired using a 2-0 Vicryl suture, followed by a full-thickness flap closure with a drain.

### Post-operative care

Immediately post-operatively, all patients were given a sling for comfort. The chest tubes were removed after the drainage decreased to less than 100 ml/day. In order to enhance the patients’ post-operative recovery, strategies such as deep breathing, coughing and early mobilization were implemented. Physical therapy was initiated two weeks after definite fixation. Full passive and active range of motion (ROM) as well as therapist-assisted stretching were allowed to regain ROM. After four weeks, the focus was placed on regaining full active ROM. All restrictions were lifted at three months. The post-operative follow-ups occurred at two and six weeks after surgery, and at three, six, and twelve months. Anteroposterior (AP) and scapula Y radiograph were taken at each clinic appointment. Malunion and nonunion of the scapula fracture were defined according to a previous study [14]: malunion-- displacement >5 mm on AP or scapula Y radiograph, persistent angulation >10° on scapula Y radiograph, or a difference≧10° in GPA compared with the contralateral shoulder; nonunion-- a persistent fracture line on any radiographic view 12 weeks postoperative, or a painful shoulder with any sign of fixation failure, including broken hardware or screw pull-out. The Disabilities of the Arm, Shoulder, and Hand (DASH) questionnaire was evaluated at each clinic appointment, except at two weeks after surgery.

## Results

**Fig. 3:**
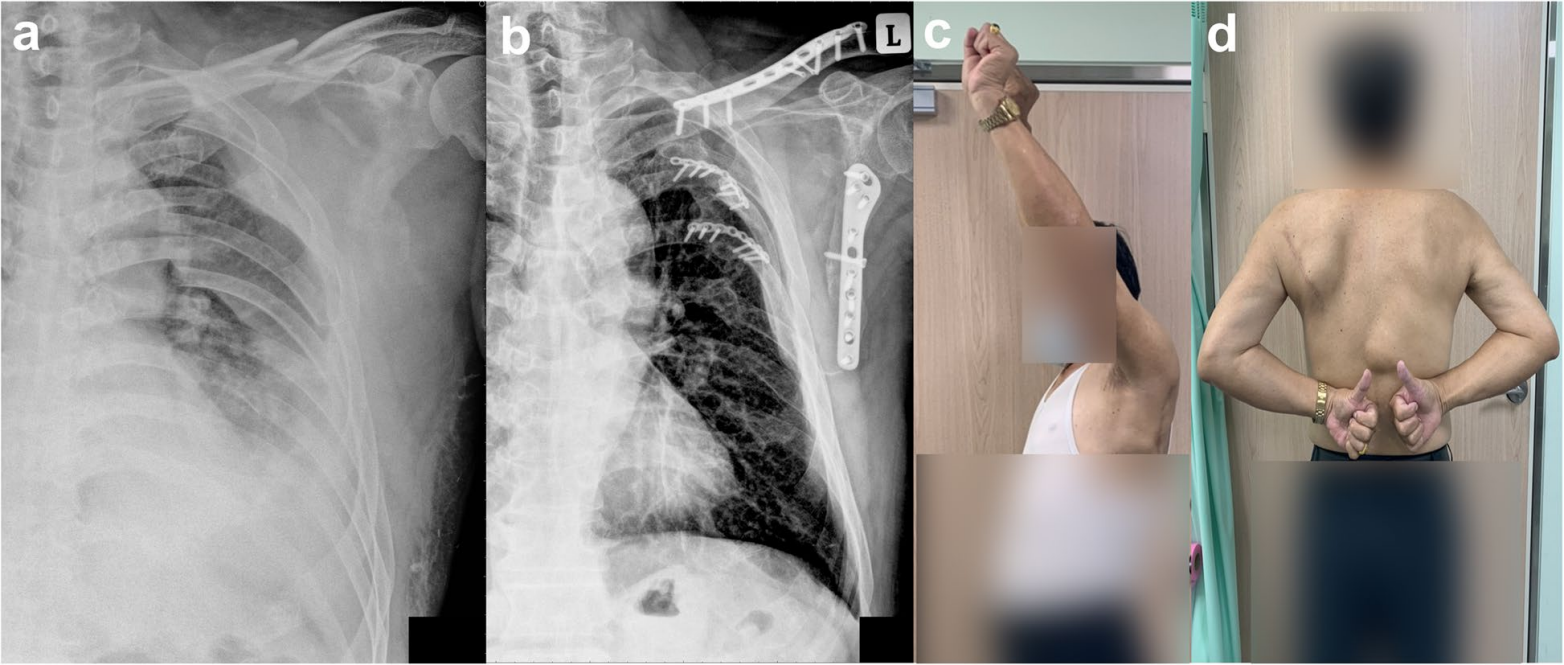
A 60-year-old male (case No. 2) presented a left scapula fracture (AO/OTA 14B(lm) type), ipsilateral clavicle and second to seventh rib fractures with hemothorax and pneumothorax (**a**). Using the mirror Judet approach, the scapula and the fourth and fifth ribs were reduced and stabilized. At the 12-month follow-up, the radiograph showed union of both the scapula and rib fractures (**b**). He had already returned to his original work with a shoulder ROM comparable with that of the other side (**c**, **d**) and three points in the Disabilities of Arm, Shoulder, and Hand score

The demographic data and surgical information of the five patients are listed in Tables 1 and 2. All patients presented surgically-indicated scapula fractures and multiple rib fractures with hemopneumothorax and associated lung contusions. After the index procedure (Table 2), all patients underwent extubation within two days after the index surgery. There was no need for regular analgesics after two weeks in all five patients. At the last follow-up, all fractures of the surgically fixed scapulas and ribs demonstrated clinical and radiographic evidence of union. There was no nonunion for all fractures and no malunion for scapula fractures (Table 1). There was no loss of reduction or signs of implant loosening. No complaints of intercostal neuralgia, infection, or other complications were seen. The mean range of motion (ROM) for the injured/uninjured shoulder was 157.8°/162.2° of forward flexion, 120.0°/124.4° of abduction, and 85.2°/86.4° of external rotation with the arm by the patient’s side and the elbow flexed to 90°. The internal rotation of the injured shoulder reached a similar level as the uninjured side. The mean DASH score was 2.0 (range: 0-7) at the twelfth month after surgery. All patients were able to return to their previous work (Fig. 3 and Table 3).

**Table 1:** Demographics and fracture characteristics

Case No.	Sex	Age	Scapular fracture classification		Rib fractures		Associated injuries	Fracture characteristics (pre-op/post-op)		
			AO/OTA	Mayo	Location	OP indication		Medial/lateral displacement (mm)	Angulation (°)	GPA (°)
1	F	44	F1.B (lm)	Type IV	2-6, posterior-lateral	Multiple ribs fx	Lung contusion, PTX,HTX	33/0	11/0	19/34
2	M	60	F0.B (lm)	N.A.	2-7, posterior	Multiple ribs fx	Clavicle fx, lung contusion, PTX, HTX	13/0	13/0	31/40
3	F	58	F0.B (lm)	N.A.	1-8, posterior	Multiple ribs fx with flail chest	Clavicle fx, lung contusion, PTX, HTX	25/0	15/0	22/35
4	M	54	F1.B (lm)	Type IV	2-8, posterior	Multiple ribs fx with flail chest	Clavicle fx, Lung contusion, PTX, HTX	14/0	7/0	33/39
5	F	56	F0.B (lm)	N.A.	3-7, posterior-lateral	Multiple ribs fx	Clavicle fx, lung contusion, PTX, HTX	22/0	8/0	15/34

*Abbreviations*: *Fx* Fracture, *PTX* Pneumothorax, *HTX* Hemothorax, *N.A.* Not applicable

**Table 2:** Surgical information

Case No.	Time to surgery (days)	EBL (ml)	Operative time (mins)	Fracture fixation		Thoracoscopy
				Scapula (lateral border + neck)	Ribs	
1	3	400	228	A-LP	5&6; L-recon (2.4 mm)	Yes
2	4	350	171	A-LP	4&5; L-recon (2.4 mm)	Yes
3	3	380	183	A-LP	4-7;L-recon (2.4 mm)	Yes
4	3	250	155	A-LP	4-6;L-recon (2.4 mm)	Yes
5	2	300	146	A-LP	4&5;L-recon (2.4 mm)	Yes

*Abbreviations*: *EBL* Estimate blood loss, *A-LP* Anatomical locking plate, *L-recon* Locking reconstruction plate

**Table 3:** Clinical follow-up outcomes

Case No.	Follow-up (months)	DASH			ROM(°) (injured/non-injured) at 12 months				Return to work
		3 month	6 month	12 month	FF	ABD	ER	IR (vertebra)	
1	18	20	8	0	160/170	160/170	78/80	T8/T6	Yes
2	20	11	6	3	152/155	95/100	68/70	T12/T11	Yes
3	12	4	1	0	158/160	132/132	74/75	T8/T8	Yes
4	14	12	8	7	165/170	146/150	72/76	T11/T10	Yes
5	12	5	0	0	156/156	138/140	74/76	T8/T7	Yes

*Abbreviations*: *DASH* The Disabilities of the Arm, Shoulder and Hand score, *ROM* Range of motion, *FF* Forward flexion, *ABD* Abduction, *ER* External rotation, *IR* Internal rotation

## Discussion

Scapula fractures occur infrequently [1], and the majority occur due to high-energy mechanisms [19]. Associated injuries are common, with chest trauma being the most common [1, 2, 20]. Rib fractures are typical chest injuries and reported with the high co-incidence of up to 52.9% [2]. Therefore, management of concomitant scapula and rib fractures are an issue. Until now, however, there remains no absolute surgical indication for rib fractures despite evidence showing the surgical benefits, including decreased risk of pneumonia, pain improvement, and increased respiratory function [9]. For scapula fractures, clearer indications have been proposed [8, 21] and excellent mid-term outcomes reported in indicated patients [21]. Therefore, a single surgical approach for the fixation of surgically-indicated scapula fractures and concomitant ipsilateral rib fractures would be helpful.

Approximately 80-90% of scapula fractures involve the neck and body of the scapula [22]; therefore, the posterior approach is the most common surgical fixation of scapula fractures. In addition to the original Judet approach [23], several surgical approaches, including the modified Judet approach [13], minimally invasive approach [14], and reverse Judet approach [15], have been proposed to minimize soft tissue trauma. However, for complex fracture patterns, the traditional Judet approach should be considered [24]. The longitudinal incision part of the mirror Judet approach is similar to the lateral border incision in the minimally invasive approach proposed by Gauger et al. [14]. Therefore, our approach was designed through the intermuscular (infraspinatus and teres minor) intervals with deltoid sparing to avoid soft tissue injury. This approach is not suggested for complex or neglected fracture patterns and indicated only in AO/OTA 14 F0.B-, F1.B-, or B-type scapula fractures whose main fixation relies on lateral border fixation with or without a simple glenoid fossa fragment. For cases that need augmented fixation in a superior medial angle (SMA) of scapula, we suggest another minimally invasive incision for SMA plating following lateral border/neck fixation. In spite of easy access to the SMA using subcutaneous dissection through the mirror Judet incision, this approach is unfavorable to the placement of the plate and screws. With proper patient selection, the mirror Judet approach can provide good visualization for fracture reduction and fixation in the scapula lateral border and lead to good functional outcomes. All five of our patients regained at least 90% of shoulder ROM compared with the contralateral side and returned to their original work and activities. There were no malunions or nonunions.

Currently, all indications for SSRF are still considered to be relative. Growing evidence has supported surgical fixation in selected patients with a flailed chest, multiple (more than three) rib fractures with bicortical displacement, and other concomitant pulmonary lesions that need surgical intervention [7, 9]. Two meta-analysis studies supported that SSRF for flailed chest significantly decreased the mortality [25, 26]. Other studies have shown decreased pain, shorter duration for ventilation support, and decreased hospital and intensive care unit stay [3, 4, 27–29]. On the other hand, no benefit to SSRF for flailed chest has also been reported [30, 31]. Accordingly, there is still no consensus for indications of SSRF and selection of ribs for fixation. Even for a flailed chest, SSRF is still not a routine procedure. Our approach is similar to the modified muscle-sparing posterolateral thoracotomy approach and allows direct access to the posterior and lateral aspects of ribs 4-8 [17], which usually are the fixation targets. There is also no study that compares the functional outcomes of patients after different approaches for SSRF. Even though the muscle-sparing technique has been shown to improve initial muscle strength in patients undergoing thoracotomy for lung resection or lobectomy, no difference was seen at one month after surgery comparing to posterolateral approach [32, 33]. Further studies to determine who would benefit from SSRF, which rib fractures require fixation, the optimal timing for SSRF, and cost-effectiveness are necessary.

There are some limitations in this study. First, only five patients were included and analyzed. Concomitant and ipsilateral scapula and multiple rib fractures that meet the surgical indications are uncommon. We propose the mirror Judet approach, which allows for surgical stabilization of both the scapula and rib fractures with the same approach aimed at minimizing soft tissue dissection. Using this approach, the interval for the lateral border of the scapula is intermuscular, and that for the posterior and lateral portion of the 4th-10th ribs is muscle sparing (muscle splitting). Our preliminary results showed acceptable clinical outcomes in well-selected patients. Second, there is no comparison group using two separate approaches for surgical stabilization of both scapula and rib fractures. Further comparative cohort studies with larger patient populations are needed to confirm the outcomes.

## Conclusion

For concomitant and ipsilateral scapula and multiple rib fractures that meet the surgical indications, the mirror Judet approach allows adequate fixation for both fractures and provides acceptable functional outcomes in well-selected patients.

## Data Availability

The datasets used and/or analysed during the current study are available from the corresponding author (PYL or PTW) on reasonable request.
